# Clear cell odontogenic carcinoma of the mandible: a treatment strategy

**DOI:** 10.1590/1678-7757-2016-0645

**Published:** 2018-01-16

**Authors:** Sabrina FERREIRA, Leonardo Perez FAVERANI, Gabriel Mulinari dos SANTOS, Everton Pontes MARTINS, Idelmo Rangel GARCIA

**Affiliations:** 1 Univ. Estadual Paulista, Faculdade de Odontologia de Araçatuba, Departamento de Cirurgia e Clínica Integrada, Araçatuba, SP, Brasil.; 2 Santa Casa de Misericórdia de Araçatuba, Araçatuba, SP, Brasil.

**Keywords:** Odontogenic carcinoma, Clear cell, Mandible

## Abstract

Clear cell odontogenic carcinoma (CCOC) is a rare odontogenic tumor of the jaws, histologically characterized by the presence of agglomerates of cells with eosinophilic cytoplasm. The patient, a 62-year-old Caucasian woman, presented an intraosseous lesion in the mandibular symphysis. A clinical examination revealed a discrete volumetric increase with a hard consistency, palpable to extraoral and intraoral examinations. Imaging studies revealed an extensive radiolucent area, without defined limits, extending from the region of the right second premolar to the left canine. Incisional biopsy analysis indicated a diagnosis of CCOC. The treatment proposed was segmental resection of the mandible with a safety margin. After six months without recurrence, definitive mandibular reconstruction was performed using an iliac crest graft, followed by rehabilitation with implant-supported denture after five months. After three years of post-resection follow-up, the patient has shown no evidence of recurrence or metastasis. She continues to be under follow-up. To conclude, CCOC must be considered a malignant tumor with aggressive behavior. Previous studies have shown that resection with free margins is a treatment with a lower rate of recurrence. Nevertheless, long-term follow-up is necessary for such patients.

## Introduction

Clear cell odontogenic carcinoma (CCOC) is a rare odontogenic tumor of the jaws, histologically characterized by the presence of agglomerates of cells with eosinophilic cytoplasm. The latest review of the English literature revealed about 87 well-documented cases of CCOC^14^.

CCOC has no specific clinical and radiographic signs, making its diagnosis difficult. The predominant histopathological characteristic is the presence of isles of cells with clear or eosinophilic cytoplasm, with well-defined outlines and nuclei in a central position^8^. Nevertheless, CCOC is not the only lesion that presents clear cells. They may also be observed in neoplasias such as calcifying epithelial odontogenic tumor or Pindborg tumor, ameloblastoma with a component of clear cells, and various odontogenic cysts^6^ ^,^ ^13^. Long-term follow-up is suggested in the literature because of its potential for recurrence and distant metastases^12^.

This study documents a case of extensive CCOC of the mandible, treated with segmental resection of the mandible, with subsequent reconstruction using an autogenous iliac crest bone graft and rehabilitation with an implant-supported denture.

## Case report

The patient, a 62-year-old Caucasian woman, visited our Oral and Maxillofacial Traumatology and Surgical service because of the chance discovery of an intraosseous lesion in the mandibular symphysis. A clinical examination revealed a discrete volumetric increase of unknown origin in the anterior region of the mandible, with a hard consistency, palpable to extraoral and intraoral examinations. The patient did not report any pain, absence of continuity solution in the soft tissues of the region, had no history of trauma and sensory changes in the region, and had non-palpable lymph nodes.

**Figure 1:**
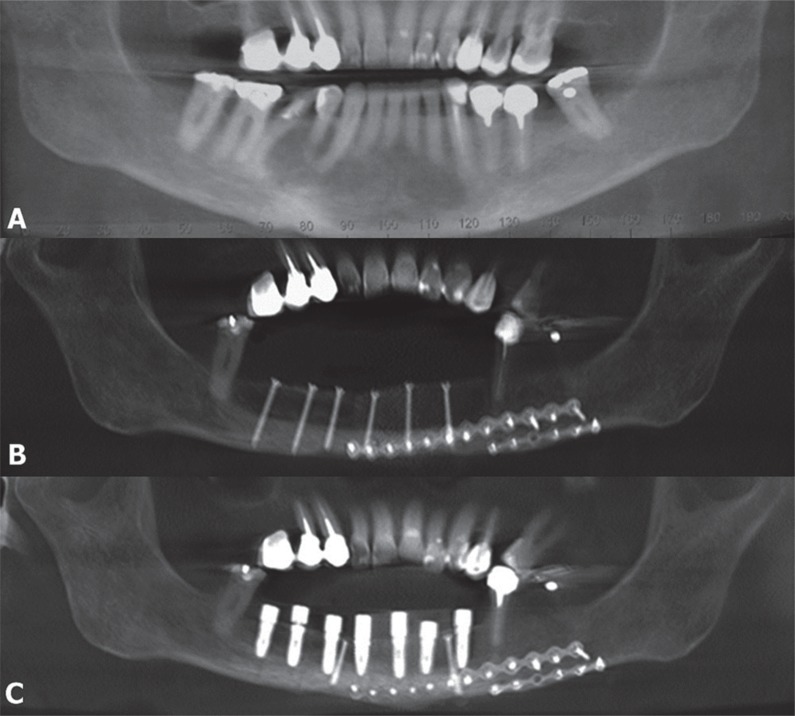
Panoramic radiographs – (A) Preoperative radiograph shows an extensive radiolucent area, without defined limits, extending from the region of the right second premolar to the left canine. (B) Panoramic radiograph showing the restoration of mandibular morphology after 6 months of follow-up; areas suggestive of recurrence are absent. (C) Panoramic radiograph showing the progress of rehabilitation with a dental implant after 5 months

The panoramic radiograph showed an extensive radiolucent area, measuring approximately 5 mm along its longest axis, without defined limits, and extending from the region of the right second premolar to the left canine (Figure 1A). Cone beam computed tomography revealed an extensive hypoattenuating area, with changes in the mandibular outline, as well as an area with perforated external cortical bone at the premolars. Incisional biopsy was performed and microscopic diagnosis confirmed a malignant neoplasm of the CCOC type.

**Figure 2:**
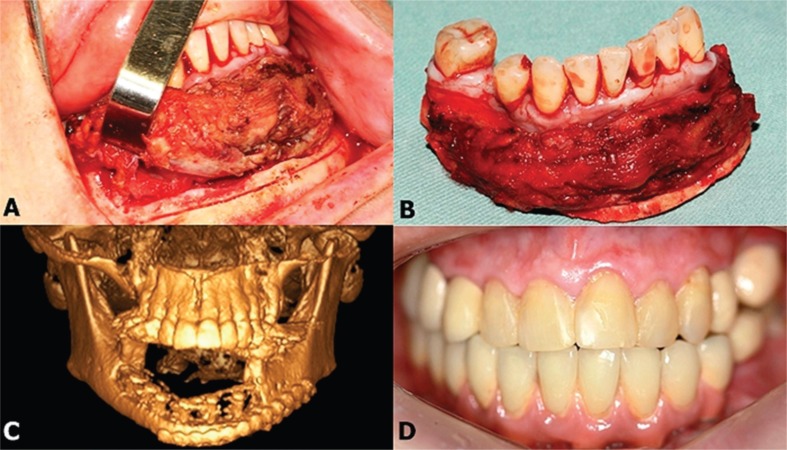
Clinical features – (A) Transoperative period showing mandibular osteotomy for tumor exeresis. (B) Tumor peace after exeresis. (C) Computadorized tomography after one day postoperative showing stability of fixation. (D) Clinical aspect after prosthesis rehabilitation

**Figure 3:**
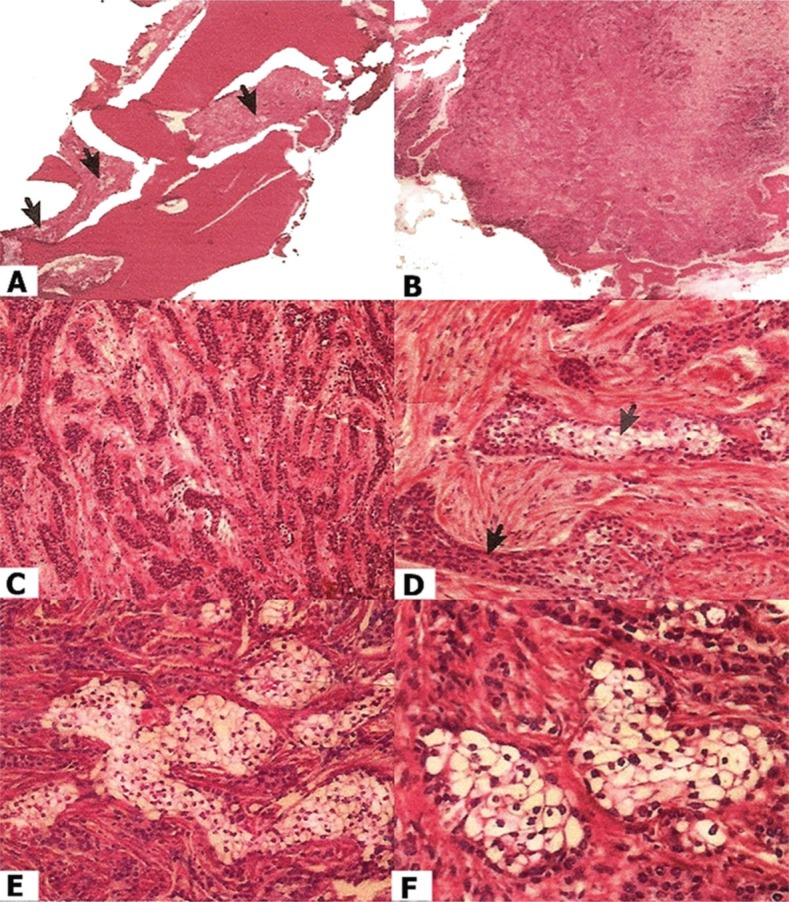
Histological features – (A, B and C) Lesion with a trabecular pattern, with moderate desmoplasia, without necrosis, showing infiltration into the trabecular spaces of the bone tissue (black arrows). (D, E and F) Presence of clear cells (black arrows) confirming tumoral trabeculae into mandible bone

The treatment proposed was segmental resection of the mandible with a safety margin under general anesthesia (Figures 2A, 2B and 2C). Histopathological analysis of the resected tissue confirmed the diagnosis of CCOC, showing evidence of a lesion with a trabecular pattern, with moderate desmoplasia, without necrosis, showing infiltration into the trabecular spaces of the bone tissue (Figures 3A, 3B and 3C; black arrows), and the presence of clear cells (Figures 3D, 3E and 3F, black arrows). Radiographs of the thorax and abdomen were also acquired with the objective of locating areas of metastases or an unknown primary tumor, but the findings were negative.

After six months of radiographic follow-up, the patient showed no signs of recurrence; hence, definitive mandibular reconstruction was performed using an autogenous iliac crest bone graft (Figure 1B). This was followed by rehabilitation with an implant-supported denture after five months (Figures 1C and 2D). After three years of post-resection follow-up, the patient showed no evidence of recurrence or metastasis, continuing to be under follow-up by the team.

## Discussion

Considered more aggressive than ameloblastoma, CCOC occurs more commonly in middle-aged women^14^. This particular case occurred in the anterior region of the mandibular bone, which, based on recent literature, is considered rare^14^; CCOC has no established pattern of occurrence, since a few years ago the predominant area was the anterior mandibular region^18^.

CCOC has no specific clinical and radiographic signs, making it difficult to diagnose. The most frequent symptoms include pain or discomfort, broadening of the mandible, mobility or displacement of teeth, and cortical destruction^5^ ^,^ ^9^ ^,^ ^13^ ^,^ ^14^ ^,^ ^19^. In this case, the patient showed no painful symptoms, which probably contributed to the progression of the lesion and its late diagnosis. Radiographic analysis showed that the lesion was unilocular, with irregular and poorly defined margins and showing evidence of bone destruction. Some authors have also mentioned a multilocular aspect^15^ ^,^ ^19^.

The diagnosis in this case was consistent with CCOC. On macroscopic analysis, the fragments had a dark-red color, firm-elastic consistency, and hardened areas, features of bone tissue. Microscopic analysis also revealed moderate desmoplasia with a trabecular pattern but without necrosis, infiltrating into the trabecular spaces of the bone tissue, as well as nests of clear cells (Figure 3).

Clear cells generally result from factors such as intracellular accumulation of colorless compounds, such as glycogen, lipids, and mucin. Clear cells may also be the result of a scarcity of cellular organelles or an artifact induced during the fixation or processing of tissues^10^. The presence of clear cells in an odontogenic neoplasm may be associated with its supposed origin from the dental lamina, which contains clear cells^1^.

However, clear cells are not exclusive to CCOC. They may be observed in numerous neoplasias of the maxilla, such as the variant of clear cells seen in calcifying epithelial odontogenic tumor, odontogenic cysts, clear cell tumors of the salivary glands, and variations of carcinoma (e.g., acinar cell carcinoma, squamous cell carcinoma, and sebaceous tumors)^9^.

Some tumors of non-odontogenic origin are also characterized by clear cells, histologically similar to those seen in CCOC, and occur in organs such as the lung, breast, kidney, thyroid gland, and colon^18^. The concern about these lesions is that their metastases may be diagnosed in the mandible. Therefore, when a patient is diagnosed with CCOC, a thorough investigation is recommended to search for metastatic lesions of clear cell primary carcinoma, particularly of renal origin^18^. In this case, computed tomography of the thorax and abdomen was performed, but lesions were not detected.

Because of the rarity of the lesion, the ideal treatment approach has not yet been conclusively determined. Mandibular resection is indicated depending on the time of recurrence as well as its aggressiveness and destructiveness^18^. However, the availability of limited data makes it difficult to formulate risk factors for tumor recurrence and metastases^18^. Moreover, the degree of nuclear pleomorphism and hyperchromatism is extremely variable, and appears to be associated with the metastatic potential of the tumor^4^ ^,^ ^16^. Another important consideration when evaluating recurrence is the presence or absence of surgical safety margins^12^.

In some cases of CCOC, in addition to resection, bilateral removal of the cervical ganglia has been indicated in mandibular lesions, even in the absence of lymphadenopathies on initial clinical examination^7^ ^,^ ^9^ ^,^ ^20^. The same observation has been made in the literature with regard to the indication for radiotherapy^21^. Unfortunately, the number of patients receiving radiotherapy has been insufficient to evaluate the benefits of these treatment modalities.

An initial presentation of metastatic lymph nodules is rare. Some authors have indicated adjuvant ganglion removal therapy^8^ and/or radiotherapy when there is evidence of extensive soft-tissue invasion, perineural invasion, or positive lymph nodes, or when removal of the tumor with free margins is not feasible^2^ ^,^ ^3^ ^,^ ^9^ ^,^ ^12^ ^,^ ^17^.

In this case, partial surgical resection of the mandible was performed with margins of healthy bone tissue. In the absence of palpable lymph nodes and metastatic lesions, ganglion removal was not performed. Radiotherapy and chemotherapy were also not indicated. The patient has been radiographically followed up for three years, without signs of recurrence or metastatic lesions. In the literature, the general rate of recurrence has been reported as 41.8% and 86.7% among patients treated with curettage, and 29.8% among those treated with resection^21^. Among the patients with recurrence, 17% had distant metastases. The time interval of disease recurrence ranged from 6 to 24 months for curettage, and 11 to 71 months for resection alone^9^.

## Conclusion

In conclusion, CCOC must be considered a malignant tumor with an aggressive behavior. Previous studies have suggested that resection with free margins is a treatment associated with a lower rate of recurrence. However, curettage or enucleation appears inadequate. Moreover, long-term follow-up is necessary for such patients.
